# Feasibility, Subjective Effectiveness, and Acceptance of Short Virtual Reality Relaxation Breaks for Immediate Perceived Stress Reduction in Emergency Physicians: Single-Arm Pre-Post Intervention Study

**DOI:** 10.2196/72605

**Published:** 2025-07-04

**Authors:** Tanja Birrenbach, Seraina Häni, Sabrina Jegerlehner, Simon Schober, Aristomenis K Exadaktylos, Thomas C Sauter

**Affiliations:** 1 Department of Emergency Medicine, Inselspital, University Hospital of Bern, University of Bern, Rosenbühlgasse 27, Bern, 3010, Switzerland, 41 316322111

**Keywords:** virtual reality, relaxation, stress, emergency medicine, workplace, burnout

## Abstract

**Background:**

Emergency physicians face significant stress in their daily work, adversely affecting patient care and contributing to physician burnout.

**Objective:**

This pilot study explored the feasibility, immediate effects, and acceptance of virtual reality (VR) relaxation on perceived stress reduction among emergency physicians.

**Methods:**

The study was conducted at the Department of Emergency Medicine, Bern, Switzerland, in February 2023. All junior and senior physicians were eligible, excluding those with epilepsy, claustrophobia, or severe nausea. Voluntary participants underwent a 6- to 8-minute VR meditation program at their workplace. Subjective short-term stress reduction was measured using a numeric rating scale (NRS) ranging from 0 (“not at all stressed”) to 10 (“extremely stressed”). Feasibility, user acceptance, and technical aspects were evaluated using validated and self-constructed questionnaires.

**Results:**

In total, 35 emergency physicians (median [IQR] age, 32 [30-34] years, 60% female) completed 39 VR simulation sessions. Baseline stress levels (median NRS 4, IQR 2‐6.5) were significantly reduced post-intervention (median NRS 2, IQR 1‐4; *P*<.001), particularly among participants with high baseline stress levels. Reported side effects (simulator sickness) were minimal; the median score of presence and immersion according to the questionnaire developed by Slater-Usoh-Steed was 4 (IQR 3‐4) (scale 1‐7, with 7=full immersion). User satisfaction was high. Implementation challenges mainly included technical issues and time constraints due to high workload.

**Conclusions:**

This pilot study suggests that brief, relaxing VR sessions may help reduce short-term perceived stress levels in emergency physicians with minimal side effects and high user satisfaction. Future studies should address implementation challenges to optimize integration with clinical workflows.

## Introduction

Emergency medicine is an inherently high-stress medical specialty due to the urgent and often severe nature of cases, which demand rapid decision-making with potentially life-altering consequences. The additional burden of shift work and disrupted circadian rhythms further exacerbates stress levels among emergency physicians. These factors contribute to a heightened risk of burnout [1-3], posttraumatic stress disorder [4], substance abuse [5], and even suicide [6]. Burnout is a syndrome conceptualized as resulting from chronic workplace stress that has not been successfully managed. It is characterized by feelings of energy depletion or exhaustion; increased mental distance from one’s job, or feelings of negativism or cynicism related to one’s job; and reduced professional efficacy. A recent Swiss investigation confirmed emergency physicians as a medical specialty at great risk for burnout. Over half of the more than 600 respondents met at least 1 criterion for burnout and reported symptoms of mild to severe depression. Alarmingly, 10% of respondents even reported having considered suicide at some point [7]. The implications of burnout extend beyond individual well-being, jeopardizing patient care quality and safety and contributing to physicians leaving the profession [7,8]. Therefore, prioritizing personal stress management strategies and advancing research into effective stress reduction methods are essential to maintaining both the quality and sustainability of emergency medicine. This aligns with the World Health Organization’s call for addressing health care worker well-being to ensure the resilience of health care systems [9].

Various stress management interventions, such as yoga, mindfulness training, deep breathing exercises, and psychoeducational stress management workshops, have demonstrated effectiveness and are increasingly being implemented in workplace settings. However, the integration of these interventions into fast-paced work environments, such as emergency medicine, remains a significant challenge [10,11].

Virtual reality (VR) is a computer-generated simulation allowing the user to fully immerse himself in an interactive, 3-dimensional environment, typically through a specialized VR headset, or head-mounted device. By blocking out the real world and replacing it with a digital space, VR allows users to engage with virtual objects and environments in real time. This immersion fosters a sense of presence, where users psychologically perceive the virtual world as real, enhancing emotional and cognitive engagement. In relaxation-focused VR applications, this heightened presence allows users to fully disconnect from external stressors, creating a safe space for restorative mental states and stress relief [12].

In the medical field, VR has long been used as a virtual therapeutic tool for managing acute and chronic pain and reducing anxiety across various settings, including the emergency department (ED) [13,14]. Additional applications include treatment for mental health conditions such as cognitive impairment, depression, phobias, and posttraumatic stress [15-17]. Research indicates that VR is an effective therapeutic tool for relaxation, modulating individual stress levels, and potential impacts on the immune response [18]. It offers a cost-effective and accessible option for therapeutic intervention [10,19-21]. Unlike traditional mindfulness practices such as meditation or yoga, VR requires little to no prior experience before positive effects can be achieved [22]. Possible explanations include the attention restoration theory, which posits that exposure to natural environments can replenish cognitive resources depleted by stress. VR can simulate calming natural scenes, providing restorative experiences that reduce mental fatigue and stress [23]. The biopsychosocial model suggests that stress is influenced by biological, psychological, and social factors. VR interventions can address these components by offering immersive experiences that promote relaxation, thereby positively affecting physiological and psychological states.

Potential barriers to the widespread adoption of VR include initial implementation and ongoing maintenance costs, limited accessibility related to hardware availability or user familiarity, uncertainty regarding the duration of beneficial effects, and the risk of adverse reactions such as visually induced motion sickness. Emerging evidence on the use of VR for health care workers suggests promising outcomes. A recent randomized controlled trial involving 32 health care workers demonstrated that VR-based guided meditations are a feasible and accessible mindfulness intervention, potentially even more effective than non-immersive methods [24]. Similarly, brief, tranquil VR experiences have been shown to significantly reduce subjective stress among frontline health care workers during the COVID-19 pandemic [25,26] and to enhance happiness and relaxation among trauma care clinicians [27].

The evidence regarding the use and effectiveness of VR as a stress reduction tool for emergency physicians remains limited. Additionally, implementing VR within the unpredictable and fast-paced environment of an ED presents significant challenges. The feasibility of its application and the acceptance by the emergency team are unclear. Therefore, we conducted a within-subject, repeated measure interventional feasibility pilot study to evaluate the feasibility of deployment of a short relaxing VR simulation in the busy setting of the ED as a stress-reduction tool for emergency physicians; the immediate effect of VR use on self-perceived stress; and the tacceptance of the VR simulation in the study population (user satisfaction, simulator sickness, and sense of presence and immersion).

## Methods

### Design and Setting

This prospective non-randomized pre-post interventional feasibility pilot study was conducted at the ED of the University Hospital of Bern, Switzerland. As one of the largest EDs in Switzerland, it serves approximately 55,000 patients annually and is staffed by a team of around 70 physicians [28]. The study was carried out between February 1 and February 28, 2023, during daytime hours, contingent on the availability of the study investigators (SH and SS).

The study was conducted on a convenient sample of emergency physicians. Written informed consent was obtained from all participants, including data anonymization and authorization for use in study analysis and publication.

### Ethical Considerations

The local ethics committee (Kantonale Ethikkommission Bern, KEK; BASEC number Req-2023‐00018) classified this study as a quality evaluation project, exempting it from the requirements of the Swiss Human Research Act.

### Inclusion and Exclusion Criteria

All junior and senior physicians working in the ED of the University Hospital in Bern were eligible for participation. Exclusion criteria included facial or neck injuries, severe nausea or vomiting, claustrophobia, epilepsy, or any other conditions associated with hypersensitivity to light or motion.

### Baseline Data

Baseline data included sociodemographic factors (gender, age), the use of visual aids, and smoking habits. Information regarding work routines was also collected, such as the participant’s role in the ED, years of professional experience, board certification, workload percentage, frequency of night shifts per month, average break duration, and typical break activities. Additionally, participants were asked about prior experience with gaming, VR, and mindfulness exercises (“I regularly use gaming, VR or mindfulness training”). The baseline questionnaire was completed before the initial use of the VR intervention.

### Intervention

Physicians were informed about the project in advance during staff meetings, and throughout the study period, reminders were provided through announcements during briefings and informational posters. Additionally, participants were recruited through direct contact by the study coordinators (SH and SS). For half of the 28 days, the study was conducted from 7 AM to 3 PM, and for the other 14 days, from 3 PM to 11 PM, corresponding to the 2 largest daily shifts. During their time at the University Hospital of Bern, the study coordinators were easily reachable via a pager system, allowing physicians to choose an appropriate time for the intervention at their discretion.

The study investigators (SH and SS) informed the participant about the study aims, handed out the information form, ensured the absence of contraindications, responded to the participant’s questions, and collected their free, informed, and expressed consent.

The intervention consisted of the application of a 6- to 8-minute VR relaxation program called “Daily Focus,” including breathing exercises and a short focus exercise in an imaginary environment. The immersive experience consists of a contemplative, relaxing, futuristic imaginary landscape accompanied by a sound universe specifically composed to relax the user. The scenery and theme changed daily. The content also had interactive capabilities as well, so that the user could take action to affect the VR environment. The user could choose to interact with the environment by fixating one’s gaze on an interactive object in the worldscape. “Daily Focus” is part of the commercially available software “TRIPP” developed by TRIPP Inc. (TRIPP Inc.). The company was not involved in any aspects of the study. A commercially available stand-alone head-mounted display (Meta Quest 2; Meta) was used. When it became apparent that background noise at the University Hospital of Bern’s workplaces impaired the sense of immersion for some participants, noise-cancelling headphones (JBL Live 650BTNC; JBL) were introduced to reduce ambient sounds. As the physicians’ experience with VR head-mounted displays was limited, the users were supported by the study team in the technical application when needed (SH and SS). In case of a medical emergency requiring the immediate presence of the physician, the VR simulation was interrupted. The briefing, completion of the consent form and questionnaires, and the intervention itself took approximately 15 minutes in total. The duration of the evaluation and intervention was intentionally kept as short as possible to minimize barriers to participation.

### Outcomes

#### Feasibility

Feasibility was assessed using technical details of the simulation (location of the simulation, ie, directy at the workplace vs quieter location, interruptions of the simulation and reasons for interruptions, and timing of the intervention), as well as with free text comments of the users and feedback collected from the study team (SH and SS).

#### Immediate Effect of VR Use on Perceived Stress

Perceived stress reduction was measured as the difference between the self-reported stress level directly before and after the intervention on a numeric rating scale (NRS-11) scale from 0 to 10 (0=”not at all stressed” to 10=”extremely stressed”). This simple measure was selected due to its strong correlation with the well-validated Perceived Stress Scale 14 (PSS-14) [29]. Furthermore, a threshold value of 6.8 on the self-reported scale has been shown to effectively predict high stress levels, corresponding to a PSS-14 cutoff score of ≥7.2, and was therefore chosen to identify individuals experiencing high stress, similar to Beverly et al [25,29].

#### User Acceptance

User acceptance was evaluated using the following questionnaires:

Visually induced motion sickness was assessed according to the Simulator Sickness Questionnaire (SSQ) from Kennedy et al [30].

Presence and immersion in the virtual world were determined according to the 6-item questionnaire developed by Slater-Usoh-Steed (total score ranges from 1=no immersion to 7=full immersion) [31].

User satisfaction was assessed using a self-constructed 8-item questionnaire (1: I enjoyed the simulation experience; 2: The headset and headphones felt comfortable; 3: The audio quality was clear and enjoyable; 4: The image quality was visually pleasing; 5: The simulation helped to reduce my stress level; 6: I would use this simulation again for relaxation; 7: I would recommend this simulation to others; 8: The simulation can be conveniently performed directly at the workplace). Responses were collected on a 5-point Likert scale (1=“totally disagree” to 5=“totally agree”) immediately following the intervention.

Furthermore, a self-constructed 6-item user acceptance questionnaire was sent out 2 weeks after the final intervention via email to all physicians working in the department, with a particular focus on understanding the limiting factors that prevented users from taking a break with VR (1: I couldn’t find time during my shift because the workload was too high; 2: I felt it wasn’t worth investing the time because I preferred to finish my documentation as early as possible to end my shift on time; 3: I didn’t enjoy the simulation (virtual environment/voice guidance), but I could imagine using it more often with a different program; 4: I experienced side effects that overshadowed the positive aspects of the VR breaks; 5: I prefer to spend my breaks differently; 6: I didn’t think about it/forgot that the option was available. Responses were collected on a 5-point Likert scale (1=“totally disagree” to 5=“totally agree”).

### Statistical Analysis

Statistical analysis was carried out using Python (version 3.9.12) and the following packages: NumPy, SciPy (matplotlib, seaborn). Baseline characteristics are presented as numbers and percentage or median and interquartile range (IQR) using descriptive statistics as appropriate. Pre- and post-simulation comparisons (stress level) were performed with the Wilcoxon signed-rank test.

We performed subgroup analyses, including participants with high stress levels defined as NRS-11 ≥6.8 (similar to Beverly et al [25]) with the Wilcoxon rank sum test.

Comparisons between independent groups (eg, male vs female, status of active patient care involvement, prior experience with mindfulness training, gaming experience) were carried out by Wilcoxon rank sum or Kruskal-Wallis test depending on the variable.

A *P*<.05 was considered significant.

Effect sizes with 95% CI for stress levels before and after the simulation were determined by Cohen *d*. Effect size was determined as follows: Cohen *d* <0.5 small, 0.5‐0.8 moderate, and >0.8 large.

## Results

### Baseline Characteristics

Out of 67 physicians (61% female), 35 working in the ED completed the study (response rate 52.2%). The average age of the participants was 32 (IQR, 30‐34) years, with 60% (n=21) being female. Further demographic characteristics as well as break behavior are reported in Table 1.

Participants were asked to rate their experience with gaming, VR, and mindfulness training (“I regularly use gaming, VR or mindfulness training”) on a scale from 1 (“Strongly disagree”) to 5 (“Strongly agree”). For gaming, the median score was 1 (IQR 1‐2), no participants had prior experience with VR, and for mindfulness training, the median score was 2 (IQR 1‐3).

**Table 1.** Baseline characteristics including break routine (N=35).

Item	Value	
Gender, n (%)		
Male	14 (40)	
Female	21 (60)	
Age in years, median (IQR)	32 (30-34)	
Use of visual aids, n (%)	16 (45.7)	
Smoker, n (%)	0 (0)	
Professional role, n (%)		
Resident physician	27 (77.1)	
Fellow physician	2 (5.7)	
Senior physician	6 (17.1)	
Board certification, n (%)	12 (34.3)	
Work experience, years, median (IQR)	5 (4-7)	
Employment level, %, median (IQR)	80 (80-100)	
Frequency of night shifts per month, median (IQR)	4 (3-5)	
Break routine, n (%)		
No breaks	6 (17.1)	
Break at the workplace with constant availability	29 (82.9)	
Break at the workplace without constant availability	0 (0)	
Average break time in minutes, median (IQR)	15 (10–20)	

### Feasibility and Technical Details of the Interventions

Out of 35 participants, 4 (11.4%) individuals completed the intervention twice, resulting in a total of 39 interventions. The majority of interventions (n=23, 59%) occurred directly at the workplace, while 41% (n=16) took place in designated rooms away from the workplace. Out of 39 interventions, 6 (15.4%) experienced interruptions. The majority of these (66.7%, n=4; 10.3% of all interventions) were due to technical issues, while the remaining 2 (33.3%, 5.1% of all interventions) were caused by urgent medical duties requiring participant attention.

In terms of shift schedules, 61.5% (n=24) of interventions were conducted during the morning shift (7–3 PM), 35.9% (n=14) during the afternoon shift (3 PM–11 PM), and 2.6% (n=1) at the end of a night shift (7 AM). During most of the interventions (n=27, 69.2%), participants remained actively engaged in patient care, whereas about one-third (n=12, 30.8%) were conducted after shift hand-over, with the participants no longer directly responsible for patient care but remaining engaged in administrative tasks.

The free-text comments were predominantly positive, highlighting the usefulness and effectiveness of the intervention. However, some criticisms were noted regarding the comfort of the headset and aspects of the simulation itself. Suggestions included a more photorealistic scenario and reduced voice guidance during the simulation. Feedback indicating that ambient emergency noises disrupted immersion was addressed by introducing the use of headphones and conducting sessions in quiet, isolated rooms whenever possible. Additionally, participants frequently mentioned that during active patient care, they were often unable to fully engage with the simulation or felt unable to allocate sufficient time for the intervention.

### Immediate Effect of VR Use on Perceived Stress Reduction

**Figure 1.**
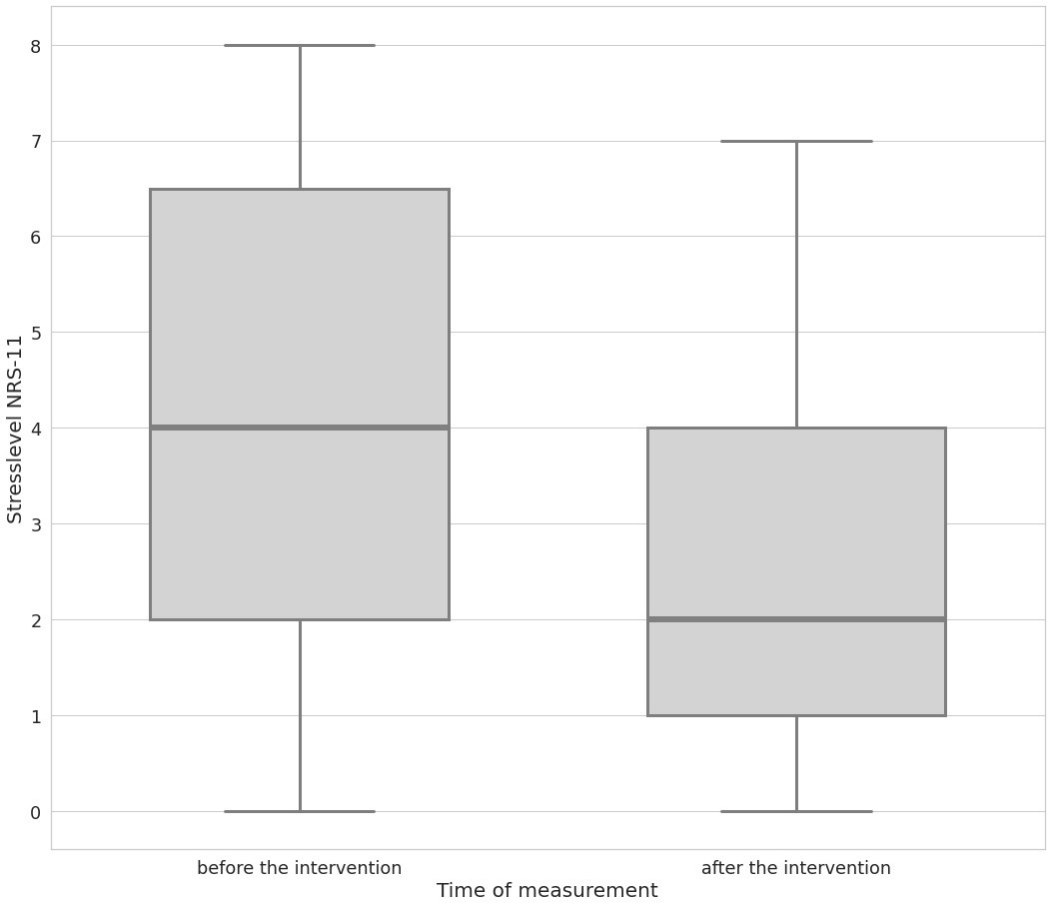
Immediate effect of virtual reality use on perceived stress reduction. Comparison of stress levels before and after the intervention. NRS: numeric rating scale.

The baseline median stress level was 4/10 (IQR 2‐6.5), which was reduced to 2/10 (IQR 1‐4) after the intervention (*P*<.001) (Figure 1). The effect size was calculated as Cohen *d*=1.28 (95% CI 0.84‐1.72), representing a large effect.

**Figure 2.**
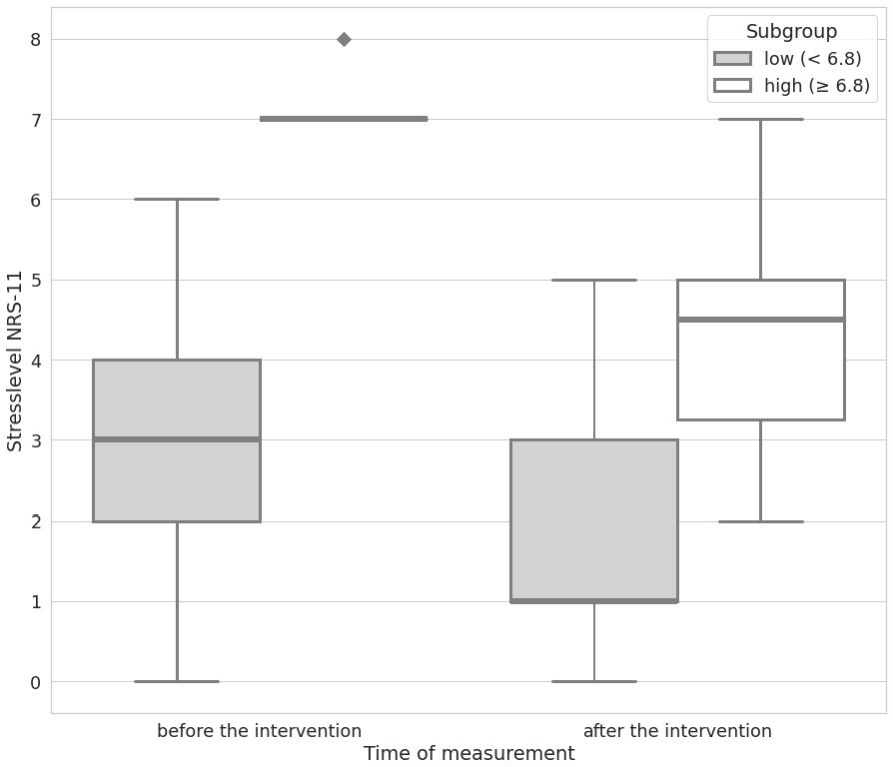
Immediate effect of virtual reality use on perceived stress reduction according to stress level. Comparison of stress levels of subgroups with low and high stress before and after the intervention. Outliers (values ≥1.5×IQR) are indicated as diamonds. NRS: numeric rating scale.

In total, 10 participants reported high baseline stress levels (≥6.8). In this group, the intervention was even more effective, reducing the stress level from 7/10 to 4.5/10 (*P*<.001) (Figure 2). Only one individual reported a high stress level after the intervention. In this case, the simulation was terminated after 2 minutes due to an audio malfunction.

No significant differences in stress reduction concerning the variables gender (*P*=.767), prior experience with mindfulness training (*P*=.376), gaming experience (*P*=.489), or involvement in active patient care (*P*=.912) were found.

### User Acceptance of the VR Simulation

#### Visually Induced Motion Sickness

The median of the total score according to the SSQ from Kennedy was 80 (IQR 0‐161) (range 0‐813).

#### Presence and Immersion

The median score of presence and immersion according to the questionnaire developed by Slater-Usoh-Steed was 4 (IQR 3‐4) (with 7=full immersion).

#### User Satisfaction

**Figure 3.**
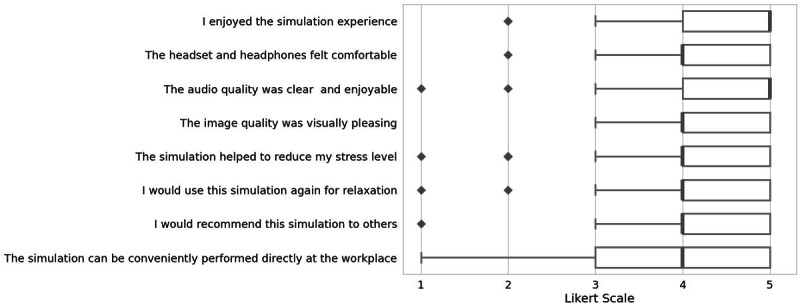
User satisfaction survey. Results of the user satisfaction survey. Answers on a 5-point Likert scale from 1=“totally disagree” to 5=“totally agree” directly after the intervention. Outliers (values ≥ 1.5×IQR) are indicated as diamonds.

Results of the user satisfaction survey are detailed in Figure 3.

#### Acceptance Survey

**Figure 4.**
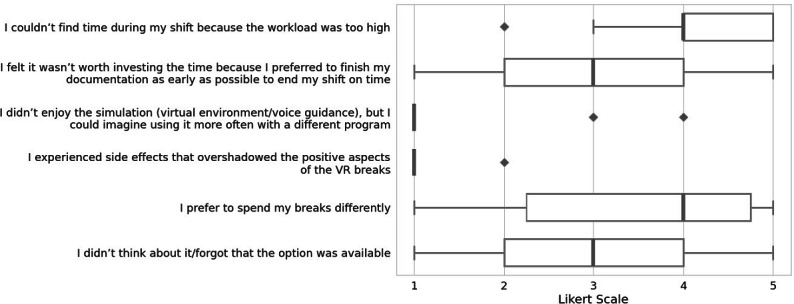
Retrospective acceptance survey. Results of the retrospective acceptance survey. Answers on a 5-point Likert scale from 1=“totally disagree” to 5=“totally agree.” Outliers (values≥1.5×IQR) are indicated as diamonds.

In total, 16 physicians completed the 6-item retrospective acceptance survey sent out 2 weeks after the intervention period (response rate 24%). Answers are depicted in Figure 4.

## Discussion

### Overview

This pilot study evaluated the feasibility, immediate effect of VR use on perceived stress reduction, and acceptance of VR simulation as a short break intervention within the high-pressure environment of an ED.

We observed a significant reduction in self-reported stress levels, decreasing from 4/10 to 2/10, with a large effect size. Importantly, 26% of participants reported high stress levels prior to the intervention, in which the stress-reducing effect of VR was particularly pronounced. No significant differences in stress reduction were observed across demographic or experiential variables, including gender, prior mindfulness training, gaming experience, or engagement in active patient care during the intervention.

Acceptance was high with minimal side effects. Despite its effectiveness, challenges were noted in implementing VR breaks, primarily due to the substantial time constraints faced by health care professionals. These logistical barriers may limit the practical application of this intervention in routine clinical practice.

### Feasibility

The simulation was carried out with minimal technical issues or interruptions. Notably, a significant number of individuals voluntarily participated in the study, and the overall feedback was highly positive, indicating strong interest and acceptance of the concept.

One limitation observed was the execution of the simulation directly in the busy ED environment at the participants’ desktops, which occasionally resulted in distracting background noise that could impact participants’ focus. To address this issue, noise-canceling headphones were introduced, and approximately 40% of the simulations were conducted in quieter settings in the ED.

### Stress Reduction

This study demonstrated a significant reduction in subjective stress levels, with an average decrease of 2 points on the NRS-11 scale following a 6- to 8-minute VR simulation. These findings align with previous studies investigating VR-based stress reduction in high-stress medical environments, particularly during the COVID-19 pandemic.

For example, Beverly et al [25] conducted a similar study involving frontline health care workers. They observed comparable reductions in stress (mean change –2.2 on a visual analogue scale from 1 to 10, effect size Cohen *d =* 1.08) and high levels of acceptance after a 3-minute 360-degree cine-VR simulation featuring a nature scene. Similarly, Putrino et al [32] reported on the effectiveness of “Recharge Rooms,” immersive multisensory environments designed to alleviate stress among frontline health care workers during the pandemic. These rooms incorporated visual projections of natural landscapes, calming sounds, and soothing scents. In a study involving 496 participants, average self-reported stress scores decreased significantly from 4.58 to 1.85 on a 6-point scale after a single 15-minute session, with high user satisfaction reported. Further supporting these findings, Nijland et al [26] evaluated the use of 10-minute VR relaxation breaks in 360° immersive environments for 86 ICU nurses during their shifts. This intervention demonstrated similar reductions in stress levels (mean change –1.4 on a visual analogue scale from 1 to 10) and high user acceptance. However, a key barrier identified across studies, consistent with our findings, was the high workload of health care professionals, which limited the feasibility of integrating VR-based interventions into routine clinical practice.

While no studies specifically targeted the ED setting, Adhyaru and Kemp [27] reported on the use of VR relaxation interventions among 39 predominately female physicians working in a fast-paced trauma service. The study highlighted the positive impact of 10-minute VR relaxation sessions using the Nature Treks application, demonstrating the potential for VR-based interventions in similar high-stress environments. Participants engaged in these sessions within a designated well-being room during their workday, immersing themselves in natural environments. Post-intervention, participants reported significant increases in feelings of happiness and relaxation, accompanied by notable decreases in sadness, anger, and anxiety. Objective measures also showed a significant reduction in heart rate, indicating decreased physiological arousal.

Although the short-term effects of various VR applications appear comparable, meaningful comparisons remain challenging due to differences in study settings, target populations, specific content and design of VR software, as well as external factors such as the surrounding environment and circumstances (eg, pandemic conditions). These variations significantly limit the generalizability and interpretability of findings across different VR studies.

Speculatively, VR’s effectiveness might be attributed to attention restoration theory, proposing that immersive restorative environments help replenish cognitive resources depleted by stress [23]. Additionally, the biopsychosocial model posits that immersive VR experiences can modulate neurophysiological responses, such as decreasing sympathetic nervous system activation and reducing cortisol levels, thereby alleviating stress [18,21].

Nevertheless, the optimal design of VR-based stress interventions remains unclear. Current literature varies widely regarding realism (naturalistic vs abstract scenarios), activity levels (passive viewing vs interactive tasks), and intervention types (guided meditation vs free exploration). These variations underline the necessity for further research using rigorous experimental designs with both cognitive and neurophysiological methodologies. Future studies should systematically investigate these variables to identify the most effective VR intervention formats and better elucidate the underlying mechanisms driving VR-induced stress reduction.

As this was a pilot study, only short-term (pre-post) effects regarding stress reduction were evaluated. However, findings from several studies provide initial data supporting the effectiveness of long-term VR-based programs for reducing stress, anxiety, and burnout among different health care professionals [24,33-36]. A recent study in the ED explored the effectiveness of a 4-week VR-based mindfulness intervention using brief guided breathing exercises. Participants using VR demonstrated greater improvements in relaxation, as measured by heart rate variability (HRV), compared to a mobile app. Regular VR use led to increased relaxation effectiveness over time, suggesting VR’s suitability for long-term mindfulness programs [24]. Several aspects require further study, such as examining patterns in VR mindfulness effectiveness across varying workload conditions and shifts. Additionally, stress and relaxation trends could be assessed by demographic or professional differences like job role or experience. It would also be valuable to explore the cumulative impact of VR sessions on chronic stress and burnout over time, analyze the timing of sessions related to well-being outcomes, and investigate how individual personality traits or baseline stress resilience influence responses to VR interventions.

### User Acceptance

With regard to side effects, the intervention proved to be largely free of adverse effects. This aligns with findings from other studies that have used VR as a relaxation tool [14,36,37].

The results indicated only moderate levels of immersion, consistent with findings from another study investigating the use of VR for pain reduction in our ED setting [14]. For both studies, we attribute this moderate immersion to environmental factors such as background noise, interruptions, and the generally high-stress atmosphere. These factors likely relate to the aforementioned limitations in implementing VR interventions within the workplace.

Overall, user satisfaction among participants was very high. Comfort, as well as the audio and visual quality, received considerable praise, particularly after the introduction of noise-canceling headphones. Participants also reported high subjective effectiveness for relaxation, with strong agreement on statements such as, “I would use this simulation again for relaxation” and “I would recommend this simulation to others.”

However, the statement “The simulation can be easily conducted in the workplace” received less agreement. This raises the question of whether and how the intervention could be better integrated into the ED workplace setting. As revealed by the retrospective questionnaire, many participants did not engage in the intervention due to work-related time pressures. This highlights a broader issue also reflected in the baseline survey results. On average, physicians reported taking only 15 minutes for breaks during their shifts.

The strong agreement with the statement “I couldn’t find time during my shift because the workload was too high” further underscores a structural challenge related to workload and break culture within the workplace. Spontaneous comments from participants and the low-medium baseline stress levels suggest that participants only took time for the intervention after the peak of their stress had passed. Given that the highest levels of work-related stress for emergency physicians typically occur during the care of critically ill patients, this timing is likely unavoidable—and perhaps even desirable. As highlighted in a recent phenomenographic study on well-being interventions in the ED, the demands of the job simultaneously necessitate and limit the implementation of effective interventions to support staff well-being in this challenging environment [38]. Possible solutions for further interventions include protected break and VR break times or scheduling VR breaks during lower workload periods.

Some participants criticized the fantasy-style design of the simulation, expressing a preference for a more naturalistic environment. However, the software used has been successfully applied in several other settings [21,34,37]. Meanwhile, many studies investigating VR for stress reduction have used realistic nature-based simulations, such as a forest. Such an approach may further enhance relaxation, as numerous studies have demonstrated that exposure to forests and nature in general promotes relaxation [10,18,20].

While we demonstrated technical feasibility and user acceptance of short VR interventions, factors such as device affordability, software licensing costs, and the scalability of deploying VR systems across various clinical settings must be carefully considered.

### Limitations

This study has several limitations that should be considered when interpreting the results. First, and mainly, the absence of a control group makes it impossible to definitively attribute the observed stress reduction to the VR intervention itself. Without a comparator, we cannot rule out alternative explanations, such as placebo effects, spontaneous recovery, or other external factors. However, given the feasibility nature of this pilot study and the promising results observed, these findings provide a solid foundation for future controlled trials. These should incorporate a more rigorous design, eg, a randomized controlled trial with a control group or an active control condition (eg, a non-VR relaxation technique, like guided breathing exercises). Second, no other structured assessments for burnout or depressive symptoms were conducted. These psychological dimensions are closely linked to stress and could have provided additional insights into the broader mental health effects of the intervention. Additionally, no physiological stress markers (eg, cortisol levels, HRV, and electrodermal skin activity) or other objective parameters were collected. Sole reliance on self-reported stress levels introduces potential biases (eg, social desirability), which may have affected the accuracy of the findings. However, as we wanted to keep the intervention as short as possible, we abstained from using an extensive test battery or setup. Future studies should include a multimodal stress assessment, potentially integrating real-time biometric data using wearable technology or mixed-reality applications. The single-center design and small sample size may also limit the generalizability of the results, as factors specific to the study setting could have influenced outcomes. Selection bias may also have influenced the results, as participants might have been particularly motivated, tech-savvy, or predisposed to respond positively to VR-based interventions. This self-selection could limit the generalizability of the findings to a broader population. The potential for a novelty effect must also be acknowledged. Participants’ stress reduction could partially stem from the excitement or novelty of using VR technology rather than the intervention’s intrinsic therapeutic effects. Furthermore, this study did not assess long-term effects. The sustainability of stress reduction over time remains unclear, and follow-up assessments would be necessary to determine whether the observed benefits persist beyond the immediate post-intervention period.

Ultimately, while the results are encouraging, future research should focus on a randomized controlled design, incorporate a multimodal assessment of stress, depression, or burnout, including objective biological stress markers, assess long-term effects, and involve larger, more diverse populations to strengthen the evidence base for VR interventions in stress management in the health care setting. Furthermore, it is essential to identify the specific aspects of the experience that elicit the most significant responses. For example, archival data before and after the Covid-19 pandemic show that passive content with less interactivity resulted in a greater positive mood state after the COVID-19 onset, likely related to its capacity to reduce stress, facilitate restoration, and improve persistent affective states in stressful environments [39].

### Conclusions

In summary, this pilot study adds to the growing evidence supporting the use of VR for workplace well-being by demonstrating the feasibility and short-term effectiveness of immersive VR simulations for stress reduction among emergency physicians. A brief VR-based relaxation break conducted directly in the ED workplace significantly decreased subjective stress levels, with high user satisfaction and minimal side effects reported. However, implementation challenges were evident, primarily due to the significant time constraints faced by health care professionals in this high-pressure environment. These findings highlight the potential of VR as a tool to enhance workplace well-being while underscoring the need for strategies to overcome logistical barriers and better integrate such interventions into routine clinical practice. Future studies should focus on long-term effects, objective stress measures, and scalable implementation strategies to further validate and optimize this approach.

## Abbreviations

ED: emergency department
IQR: interquartile range
NRS: numeric rating scale
PSS-14: Perceived Stress Scale 14
SSQ: Simulator Sickness Questionnaire
VR: virtual reality
